# The Predisposition, Infection, Response and Organ Failure (Piro) Sepsis Classification System: Results of Hospital Mortality Using a Novel Concept and Methodological Approach

**DOI:** 10.1371/journal.pone.0053885

**Published:** 2013-01-18

**Authors:** Cristina Granja, Pedro Póvoa, Cristina Lobo, Armando Teixeira-Pinto, António Carneiro, Altamiro Costa-Pereira

**Affiliations:** 1 Department of Health Information and Decision Sciences, Faculty of Medicine of Porto, Porto, Portugal; 2 CINTESIS - Center for Research in Health Technologies and Health Systems, Faculty of Medicine of Porto, Porto, Portugal; 3 Polyvalent Intensive Care Unit, São Francisco Xavier Hospital – CHLO, Lisbon, Portugal; 4 CEDOC, Faculty of Medical Sciences, New University of Lisbon, Lisbon, Portugal; 5 Screening and Test Evaluation Program, School of Public Health, University of Sydney, Sydney, Australia; 6 Intensive Care Unit, Hospital da Arrábida, Vila Nova de Gaia, Portugal; D'or Institute of Research and Education, Brazil

## Abstract

**Introduction:**

PIRO is a conceptual classification system in which a number of demographic, clinical, biological and laboratory variables are used to stratify patients with sepsis in categories with different outcomes, including mortality rates.

**Objectives:**

To identify variables to be included in each component of PIRO aiming to improve the hospital mortality prediction.

**Methods:**

Patients were selected from the Portuguese ICU-admitted community-acquired sepsis study (SACiUCI). Variables concerning the R and O component included repeated measurements along the first five days in ICU stay. The trends of these variables were summarized as the initial value at day 1 (D1) and the slope of the tendency during the five days, using a linear mixed model. Logistic regression models were built to assess the best set of covariates that predicted hospital mortality.

**Results:**

A total of 891 patients (age 60±17 years, 64% men, 38% hospital mortality) were studied. Factors significantly associated with mortality for P component were gender, age, chronic liver failure, chronic renal failure and metastatic cancer; for I component were positive blood cultures, guideline concordant antibiotic therapy and health-care associated sepsis; for R component were C-reactive protein slope, D1 heart rate, heart rate slope, D1 neutrophils and neutrophils slope; for O component were D1 serum lactate, serum lactate slope, D1 SOFA and SOFA slope. The relative weight of each component of PIRO was calculated. The combination of these four results into a single-value predictor of hospital mortality presented an AUC-ROC 0.84 (IC_95%_:0.81–0.87) and a test of goodness-of-fit (Hosmer and Lemeshow) of p = 0.368.

**Conclusions:**

We identified specific variables associated with each of the four components of PIRO, including biomarkers and a dynamic view of the patient daily clinical course. This novel approach to PIRO concept and overall score can be a better predictor of mortality for patients with community-acquired sepsis admitted to ICUs.

## Introduction

Sepsis represents a substantial health care burden [1], [2] and its incidence is increasing, in particular due to progressive aging of the population [3], [4], [5], [6], [7].

Severe sepsis and septic shock are associated with significant degrees of organ dysfunction/failure. The sequential organ failure assessment (SOFA) [8] and multiple organ dysfunction score (MODS) [9] are among the scoring systems most commonly used to describe organ dysfunction in the intensive care unit (ICU). In 2001, the North American and European sepsis definitions conference [10] convene a meeting to evaluate opinions about robustness of the existing severe sepsis criteria. The PIRO concept, which describes septic patients across four domains, aroused on that conference with the suggestion that sepsis could be looked in a similar way to cancer, with the TNM staging system. This system suggested that patients could be stratified on the basis of their predisposing conditions, the nature and extent of the insult (infection), the nature and magnitude of the host response, and the degree of organ dysfunction.

Describing sepsis-associated organ dysfunction in light of the PIRO system and introducing it in everyday practice has been challenging. The PIRO system was not assessed in any representative population until 2008, when Moreno et al [11] segregated sepsis data from the SAPS 3 database [12], and investigated whether a modified PIRO concept could be used to predict mortality in patients with infection or sepsis in the ICU. Subsequently, Rubulotta et al [13] similarly developed a score based on the PIRO concept, using two large databases of patients with severe sepsis—placebo arm patients from the PROtein C Worldwide Evaluation in Severe Sepsis (PROWESS) [14] and patients included in the PROmoting Global Research Excellence in Severe Sepsis (PROGRESS) registry [15]. Most recently, Howell et al [16] analyzed data from three observational cohorts of patients with clinically suspected infection in two U.S. centers. In a derivation cohort, a multivariable regression identified 17 covariates that were associated with hospital mortality. Similar studies have been conducted in more specific groups of septic patients [17], [18]. Although these models [11], [13], [16] have been shown to predict mortality, the variables included differed widely from one study to another, and several limitations from the different studies have introduced flaws in the diverse models [19].

Calls to dynamic views concerning sepsis staging are arising [20] and new methodological approaches have been suggested in an attempt to solve those limitations; sepsis is a dynamic process, as thus, the assessment of patterns of variation in organ dysfunctions [21] and biomarkers may be useful to assess individual outcomes in sepsis [22]. Examples of possible directions using a PIRO-based rationale are available in recent studies in which sequential changes in inflammatory markers can be surrogates of response to therapy [23] and may potentially help guide optimal duration of antibiotic therapy [24].

The Portuguese Community-Acquired Sepsis study (Sepsis Adquirida na Comunidade e internada em Unidade de Cuidados Intensivos - SACiUCI) [25], [26], [27], was designed to characterize the epidemiology of community-acquired sepsis in patients admitted to Portuguese ICUs and, in addition, to assess the level of compliance with Surviving Sepsis Campaign recommendations [26].

The aim of this study is to identify for each component of PIRO the specific variables associated with higher ability to predict hospital mortality, including a dynamic assessment of variables of the PIRO classification system.

## Materials and Methods

### Study Design

The SACiUCI study is a prospective, multi center, observational study designed to evaluate the epidemiology of community-acquired sepsis in patients who were admitted in Portuguese ICUs and has been described elsewhere [25], [26], [27].

### Definitions

Definitions for infection, community-acquired sepsis (CAS), sepsis, severe sepsis, septic shock, emergency surgery, primary admission diagnosis, primary infection source, were the same used in previous studies [25], [27]. Health-care associated sepsis (HCAS) was defined, as in our previous study [27], at hospital admission according to the presence of the following criteria: home infusion therapy (including antibiotics) or home wound care; chronic dialysis or chemotherapy within 30 days; hospitalization for 2 days or more in the preceding 90 days; residence in a nursing home or extended care facility [28], [29].

In addition, definitions for underlying disease were those from previous studies [26] and included metastatic cancer, hematological malignancy and AIDS were those used in the Simplified Acute Physiological Score (SAPS) II definitions [30]; cirrhosis, chronic heart failure, chronic respiratory failure using Acute Physiology and Chronic Health Evaluation II definitions [31]; chronic renal failure if there was need of chronic renal support or history of chronic renal insufficiency with a serum creatinine level over 2 mg/dl); HIV status (without complications defining AIDS); hematological disease including chronic neutropenia (≥3 months) or ≤1000 neutrophils/mm^3^; immunocompromised state was defined by either administration in the 12 months prior to ICU admission of chemotherapy, radiation therapy or the equivalent to 0.2 mg/Kg/day prednisolone for at least three months or 1 mg/Kg/day for a week within in the three months prior to ICU admission. Multidrug resistant (MDR) microorganisms were defined as microorganisms that were resistant to more than two different antibiotic classes and guideline concordant antibiotic therapy was considered if it was prescribed according to published guidelines for the treatment of each focus of community-acquired infection [32], [33], [34], [35], [36], [37].

### Selection of variables for each component of PIRO

The initial study protocol did not specify a classification of the variables based on the PIRO components. Therefore the authors established a classification of the variables according to the following rational:

For the P component we selected demographic variables (sex, age in years), underlying disease variables (presence of chronic failure such as hepatic, renal, cardiovascular, respiratory and hematological failure), metastatic cancer, immunocompromised state (short and long course corticosteroid therapy, chemotherapy, radiotherapy, HIV, AIDS) and number of comorbidities.
For the I component we selected variables that characterized the infection and adequacy of the initial treatment, namely type of microorganism (Gram positive, Gram negative, fungi, other, non-isolated microorganism), infection focus (urological, respiratory, neurological, intra-abdominal, other), positive blood cultures, antibiotic therapy (guideline concordant or not), multi-drug resistant (MDR), polymicrobial infection and HCAS.
For R component we considered variables that are potential indicators of response to the infection: the selected variables were C-reactive protein (CRP) (mg/dL), heart rate (bpm), white cell count (WCC) (10−3*L) and neutrophils (%).
Finally, for the O component the selected variables were those that reflect organ dysfunction namely glycemia (mg/dL), serum lactate (mmol/L) and SOFA score. Hypoglycemia was defined as glycemia lower than 90 mg/dL and hyperglycemia was defined as higher than 150 mg/dL.

Several variables included in the R and O component had repeated measurements along the first five days of ICU stay. These repeated measurements were summarized with the estimated value at day 1 and the slope over the five days according to the methodology described below.

### Statistical Analysis

We started with a univariate analysis of the data. Each variable of the PIRO components was associated with hospital discharge status (death or alive) using t-tests and Mann-Whitney tests for continuous variables and Chi-square tests for categorical variables. Variables with a p-value <0.2 were screened for the multivariable analysis.

Variables with repeated measurements over the first five days (CRP, heart rate, WCC and neutrophils for the R component, glycemia, serum lactate and SOFA for the O component) were summarized using two parameters: the estimated initial value of the measurement and the slope of the linear trend for the following days. These parameters were obtained by fitting linear mixed models, considering the repeated measurements as the outcomes and patient-specific random intercepts and random slopes for the measurement day. The best linear unbiased predictors (BLUPs in the mixed models literature) were obtained for the random coefficients (intercept as the estimate for the initial value and slope as the estimate of the trend over the five days) and were used as the summary of the variable profile for each patient. Intuitively, the idea is to fit for each patient a linear regression to his five measurements and use the intercept and slope to describe the evolution of the variable for that patient over the five days period. However, with the linear mixed models, we estimate the individual linear regressions for the patients all at the same time. Neutrophils, glycemia and serum lactate were log transformed and CRP, SOFA and WCC were square root transformed due to their skewed distribution. Therefore, the odds ratios presented for these variable refer to the transformed scales and, when indicated, they represent the increase in the odds for a 0.1 increase on the transformed scale. Given the non-linearity of these transformations it is not feasible to back transform the odds ratios to the original scales.

In the multivariable analysis, we built multiple logistic regressions for each component of PIRO to obtain the set of covariates that best predicted hospital mortality. Variables with p-value <0.2 in the univariate analysis were candidates for the final models and were kept in the models if p-value <0.1. Interactions were tested with the final group of variables but none was found to contribute significantly. Receiver operating characteristic (ROC) curves were used to assess models discrimination and the Hosmer-Lemeshow test to analyze the goodness-of-fit.

After obtaining the four logistic models for the components of PIRO, we computed the predicted probability of death for each patient according to each component. These four results were then combined in another logistic regression to obtain the overall discrimination ability for the combined components.

PASW (SPSS)® software 18.0 for MAC (SPSS, Chicago, IL, USA) and R 2.15.0 (Development Core Team. R: A Language and Environment for Statistical Computing. Vienna, Austria: 2005) were used for the statistical analysis.

## Results

### Characteristics of the study population

Participating ICUs and patients have been described previously and included 17 ICUs and 4142 patients [25], [26], [27]. A total of 891 patients with CAS were segregated from the original population and included for analysis, 206 patients were classified as having HCAS.

Hospital mortality was significantly higher in those with HCAS (46% vs. 35%, p = 0.005), with fungal infections (65% vs. 40%, p = 0.042), with positive blood cultures (46% vs. 35%, p = 0.015), with MDR microorganisms (42% vs. 11%, p = 0.047) and with guideline non-concordant antibiotic therapy (43% vs. 36%, p = 0.034). There were no significant differences in mortality according to admission diagnosis, infection focus, Gram negative or Gram positive microorganisms and polymicrobial infection (Table 1).

**Table 1:** Demographic and clinical characteristics of the study population.

	Total(n = 891)		Non-survivors(n = 337)		Survivors(n = 554)		p-value*
**Age** (years), mean±SD	60±17		65±16		58±18		<0.001**
**Age**							
<60	379	115	(30)	264	(70)		
60–80	427	171	(40)	256	(60)	<0.001	
>80	85	51	(60)	34	(40)		
**Sex**, Male	574	230	(40)	344	(60)	0.063	
**SAPS II**, mean±SD	50±19		60±20		44±15		<0.001**
**HCAS, n (%)**	206	95	(46)	111	(54)	0.005	
**Admission diagnosis, n (%)**							
Medical	703		276	(39)	427	(61)	0.087
Non-Medical	188		61	(32)	127	(68)	
**Positive blood cultures, n (%)**	158		72	(46)	86	(54)	0.015
**Antibiotic therapy, n (%)**							
Guideline non-concordant antibiotic therapy	277		120	(43)	157	(57)	0.034
Guideline concordant antibiotic therapy	562		201	(36)	361	(64)	
**MDR, n (%)**	68		38	(56)	30	(44)	0.006
**Isolated microorganism, n (%)**	361		148	(41)	213	(59)	0.107
**Multiple microorganisms, n (%)**							
None	530		189	(36)	341	(64)	
Single agent	300		119	(40)	181	(60)	0.140
Multiple microorganisms (>1)	61		29	(48)	32	(52)	
**Microorganism n (%)**							
Gram positive	185		78	(42)	107	(58)	0.244
Gram negative	171		64	(37)	107	(63)	0.113
Fungal	17		11	(65)	6	(35)	0.032
Other	31		15	(48)	16	(52)	0.184
**Infection focus, n (%)**							
Respiratory	541		201	(37)	340	(63)	0.609
Intra – Abdominal	159		69	(43)	90	(57)	0.110
Urological	63		18	(29)	45	(71)	0.116
Neurological	34		12	(35)	22	(65)	0.757
Other	94		37	(39)	57	(61)	0.745

HCAS – Health-care associated sepsis; SAPS II – Simplified Acute Physiology Score II; MDR –Multi-drug resistant.

microorganism;

* Chi-square test,

** t Student test,

IQR – interquartile range, SD – standard deviation.

### PIRO Components

#### P component

**Figure 1:**
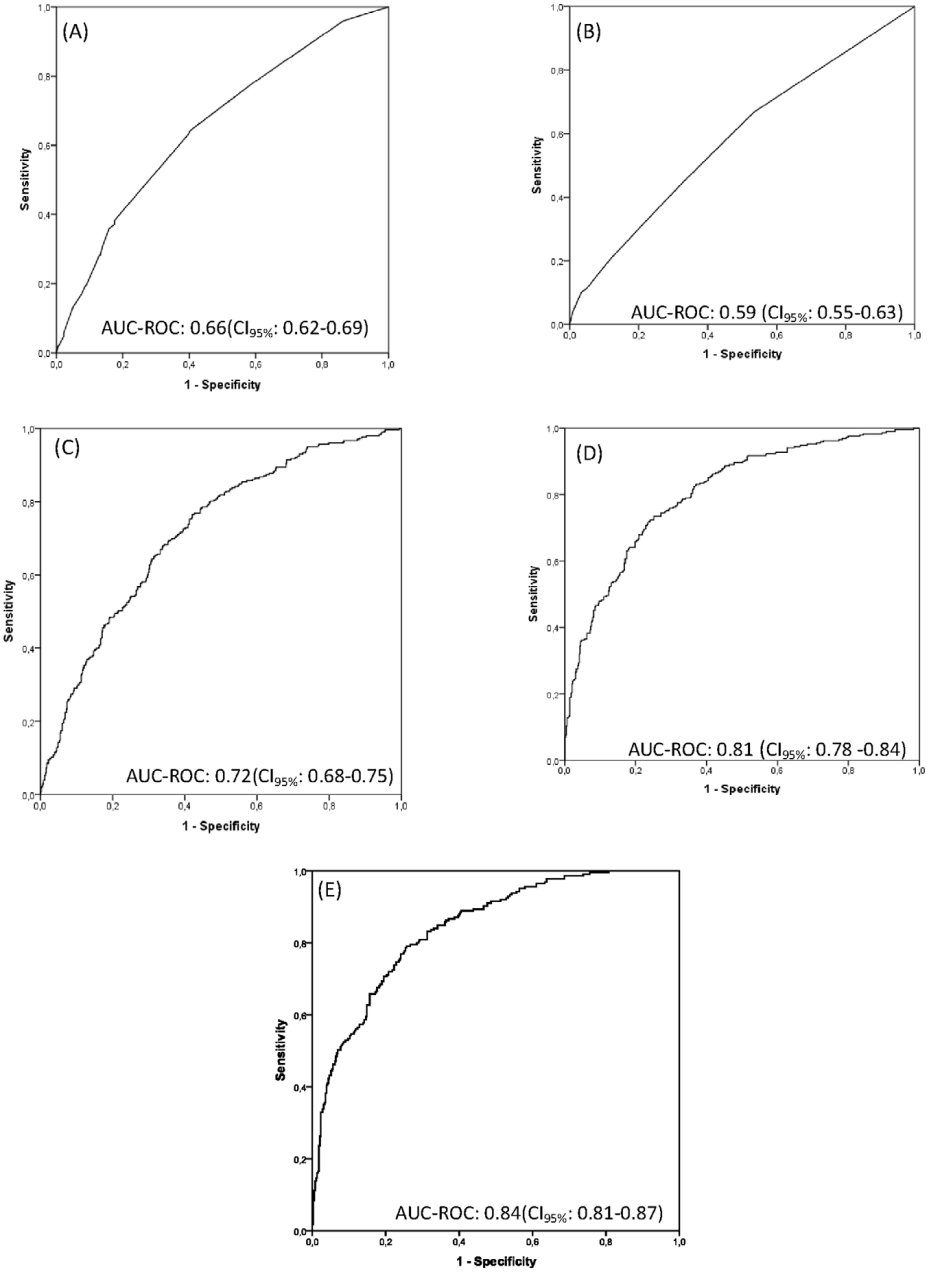
Predicted probability of death for each component of PIRO and for the combination of the four components. Receiver operating characteristic (ROC) curves and areas under the curves (AUC) for each PIRO components, Predisposition (A), Infection (B), Response (C), Organ failure (D) and the four components of PIRO combined (E).

Variables significantly associated with mortality concerning P component in univariate analysis were age (OR = 1.5 for age group 60–80, p<0.001 and OR = 3.4 for age group >80, p<0.001), chemotherapy (OR = 2.1, p = 0.025), chronic liver failure (OR = 2.1, p = 0.003), chronic renal failure (OR = 1.8, p = 0.038), chronic hematological disease (OR = 2.7, p = 0.027) and metastatic cancer (OR = 2.9, p = 0.001) (Table 2). The number of associated comorbidities was also significantly related with mortality (OR = 1.9 for 2 or more comorbidities versus none, p<0.001). In the multivariable regression analysis predisposing factors associated with mortality were gender (OR = 1.4 for male group, p = 0.025), age (OR = 1.6 for age between 60 and 80 years and OR = 4.1 for age higher than 80 years, p = 0.002 and p<0.001, respectively), chronic liver failure (OR = 2.3, p = 0.001), chronic renal failure (OR = 2.1, p = 0.007) and metastatic cancer (OR = 2.9, p = 0.001) (Table 2). Those P component variables were found to be the best predictors of death with an AUC-ROC 0.66 (CI_95%_: 0.62–0.69) (Fig. 1 (A)) and a test of goodness-of-fit (Hosmer and Lemeshow) of p = 0.297.

**Table 2:** Predisposing variables and their association with hospital mortality.

	Raw OR	p-value	Adjusted OR	p-value
**Sex, male**	1.3	0.063	1.4	0.025
**Age**				
<60	-Ref-		-Ref-	
60–80	1.5	0.004	1.6	0.002
>80	3.4	<0.001	4.1	<0.001
**Long corticosteroid therapy**	1.2	0.569		
**Short corticosteroid therapy**	1.1	0.871		
**Chemotherapy**	2.1	0.025		
**Radiotherapy**	2.0	0.259		
**HIV**	1.2	0.456		
**AIDS**	1.2	0.673		
**Chronic Hepatic failure**	2.1	0.003	2.3	0.001
**Chronic Renal failure**	1.8	0.038	2.1	0.007
**Chronic Cardiovascular failure**	1.3	0.203		
**Chronic Respiratory failure**	1.1	0.479		
**Chronic Hematological disease**	2.7	0.027		
**Cancer Disease**	2.9	0.001	2.9	0.001
**Number of co-morbidities**				
0	- Ref -			
1	1.3	0.091		
2 or more	1.9	<0.001		

HCAS - Health-care associated sepsis; HIV - Human immunodeficiency virus, AIDS - Acquired immunodeficiency syndrome, Long corticosteroid therapy - At least 0.2 mg/kg/day of prednisolone for at least 3 months in 12 months previous the hospital admission; Short corticosteroid therapy - at least 1 mg/kg/day of prednisolone for at least 1 week during the 3 months previous to the hospital admission; OR-Odds ratio.

Results from the univariate (raw OR) and multivariable (adjusted OR) logistic regressions based on a sample of 891 patients admitted to ICU with the diagnosis of community-acquired Sepsis.

#### I component

Variables significantly associated with mortality concerning I component in univariate analysis were fungal infections (OR = 2.8, p = 0.050), positive blood cultures (OR = 1.5, p = 0.015), guideline concordant antibiotic therapy (OR = 0.7, p = 0.034) and HCAS (OR = 1.6, p = 0.005) (Table 3). In the multivariable regression analysis, infection factors associated with mortality were positive blood cultures (OR = 1.7, p = 0.005), guideline concordant antibiotic therapy (OR = 0.7, p = 0.048) and HCAS (OR = 1.6, p = 0.005) (Table 3). Those I components were found to be the best predictor of death with an AUC-ROC 0.59 (CI95%: 0.55–0.63) (Fig.-1 (B)) and a test of goodness-of-fit (Hosmer and Lemeshow) of p = 0.630.

**Table 3:** Infection variables and their association with hospital mortality.

	Raw OR	p-value	Adjusted OR	p-value
**Microorganism**				
Gram positive	1.1	0.645		
Gram negative	0.8	0.191		
Fungal	2.8	0.05		
Other	1.4	0.383		
Non-Isolated microorganism	0.8	0.107		
**Infection Focus**				
Urological	0.6	0.119		
Respiratory	0.9	0.609		
Neurological	0.9	0.757		
Intra - Abdominal	1.3	0.111		
Other	1.1	0.745		
**Positive blood cultures**	1.5	0.015	1.7	0.005
**Antibiotic therapy**				
Guideline nonconcordant antibiotic therapy	-Ref-		-Ref-	
Guideline concordant antibiotic therapy	0.7	0.034	0.7	0.048
**MDR**	0.2	0.101		
**Multiple microorganisms**				
No				
1	1.2	0.251		
>1	1.6	0.071		
**HCAS**	1.6	0.005	1.6	0.005

OR-Odds ratio, HCAS – Health-care associated sepsis.

Results from the univariate (raw OR) and multivariable (adjusted OR) logistic regressions based on a sample of 891 patients admitted to ICU with the diagnosis of community-acquired Sepsis.

#### R component

Response variables significantly associated with mortality in univariate analysis were heart rate and neutrophils and CRP (Table 4).

**Table 4:** Response variables and their association with hospital mortality.

	Raw OR	p-value	Adjusted OR	p-value
Initial CRP response (squared rooted)	0.99	0.809		
CRP slope (squared rooted)	2.7	<0.001	2.3	0.001
Initial heart rate response	1.02	<0.001	1.04	<0.001
Heart rate slope	1.00	0.849	1.1	<0.001
Initial WCC response (squared rooted)	1.03	0.731		
WCC slope (squared rooted)	1.6	0.185		
Initial Neutrophils response* (log transformed)	1.00	0.396	1.04	<0.001
Neutrophils slope* (log transformed)	1.16	<0.001	1.31	<0.001

OR– odds ratio; CRP – C-reactive protein (mg/dL); WCC – white cell count (10^−3^ L); log – logarithm,

* per increase of 0.1 units.

Results from the univariate (raw OR) and multivariable (adjusted OR) logistic regressions based on a sample of 891 patients admitted to ICU with the diagnosis of community-acquired Sepsis.

Response variables associated with mortality were the slope of CRP squared root transformed (OR = 2.3, p = 0.001), initial value of heart rate (OR = 1.04, p<0.001), heart rate slope (OR = 1.1, p<0.001), initial value of neutrophils in the log scale (OR = 1.04, p<0.001) and neutrophils slope in the log scale (OR = 1.31, p<0.001) (Table 4). Those variables were found to be the best predictors of death with an AUC-ROC 0.72 (CI_95%_: 0.68–0.75) (Fig. 1 (C)) and a test of goodness-of-fit (Hosmer and Lemeshow) of p = 0.678.

#### O component

Organ dysfunction variables significantly associated with mortality in univariate analysis were total SOFA and each component of the SOFA score, with the exception of hepatic SOFA which was significant only by the third day (Table S1). Serum lactate >2 mmol/L was also significantly associated with mortality and hypoglycemia was significantly associated with mortality on day 1 and 2 and hyperglycemia on day 3 to 5.

In the multivariable model, the variables associated with mortality were initial value of serum lactate in the log scale (OR = 1.2 per increase of 0.1 units, p<0.001), serum lactate slope in the log scale (OR = 2.4 per increase of 0.1 units, p<0.001), initial value of SOFA squared root transformed (OR = 1.1 per increase of 0.1 units, p<0.001) and SOFA slope squared root transformed (OR = 2.6 per increase of 0.1 units, p<0.001) (Table 5). Those variables were found to be the best predictors of death with an AUC-ROC 0.81 (CI_95%_:0.78–0.84) (Fig. 1 (D)) and a test of goodness-of-fit (Hosmer and Lemeshow) of p = 0.588. Additionally, for the O component, we fitted the final model substituting the SOFA score by the 6 SOFA components (cardiovascular, respiratory, renal, neurological, hematological and hepatic). However, the AUC-ROC did not improve (0.81, CI_95%_: 0.78–0.84) therefore we maintained the more parsimonious model with the total SOFA.

**Table 5:** Organ variables and their association with hospital mortality.

	Raw OR	p-value	OR Adjusted	p-value
Initial Glycemia response (log transformed)	0.77	0.305		
Glycemia slope (log transformed)	24.2	0.045		
Initial Serum Lactate response (log transformed)*	1.1	<0.001	1.2	<0.001
Serum Lactate slope (log transformed)*	0.7	<0.001	2.4	<0.001
Initial SOFA response (squared rooted)	1.1	<0.001	1.1	<0.001
SOFA slope (squared rooted)*	2.3	<0.001	2.6	<0.001

OR – odds ratio; log – logarithm; SOFA – sequential organ failure assessment score,

* per increase of 0.1 units.

Results from the univariate (raw OR) and multivariable (adjusted OR) logistic regressions based on a sample of 891 patients admitted to ICU with the diagnosis of community-acquired Sepsis.

### Overall PIRO performance

The combination of the four results from PIRO's components in a single logistic regression to model the probability of death showed that each component was independently associated with mortality (p<0.001 for the P component, p = 0.004 for the I component, p = 0.002 for the R component and p<0.001 for the O component) and resulted in an AUC-ROC of 0.84 (CI_95%_: 0.81–0.87) in predicting hospital outcome. This AUC-ROC was significantly higher (p<0.001) than the AUC-ROC for SAPS II 0.74 (CI_95%_:0.70–0.77) (Fig. 1 (E)) obtained for the patients in our study.

## Discussion

This study identified a group of variables associated with each component of the PIRO staging system that are good predictors of hospital mortality for patients with CAS admitted to ICU.

Variables associated with response and organ-dysfunction took into account also their evolution over the first five days of ICU stay rather than as isolated measurements in each day. We summarize the dynamics of the measured variables with the initial value and how fast the variable changes over time (slope). These features seem to be more important than point-wise difference at specific days. This methodology was previously described concerning the same cohort of patients [27] and it seems a suitable approach for the PIRO staging system. Interestingly, it is in accordance to what has been recently suggested as a more appropriate approach [20]. It is worthwhile to highlight why this dynamic perspective may be more suitable when trying to stage sepsis patients; sepsis patients are not in a static condition but are markedly dynamic, as thus, changes overtime are much more informative of the clinical course than a single “picture” at the day of infection diagnosis or ICU admission. There are a number of variables that may not be available at the time of infection diagnosis or ICU admission but, when available, will change the treatment approach. Our perception of the patients is not determined only by the first evaluation but it changes over time with the clinical course and information coming from several diagnostic procedures (laboratory, radiology). Besides, to our knowledge, this is the first study to introduce biomarkers, namely CRP, as well as a dynamic approach to the PIRO staging system and we believe it may enhance its predictive accuracy concerning mortality, as the combination of the four PIRO's components presented very good discriminatory ability (AUC 0.85, CI_95%_: 0.82–0.88).

Moreover, we were able to confirm findings from previous studies concerning risk factors and procedures associated with an increase of mortality in sepsis patients: HCAS, higher severity of disease, fungal infections, positive blood cultures, MDR microorganisms and guideline non-concordant antibiotic therapy. Five prior studies, already mentioned, have been published addressing the PIRO concept: Moreno et al [11] included variables from 2628 ICU sepsis patients in their model that were stratified into only three components, instead of the four original components of the PIRO system; this model showed fair ability to predict in-hospital mortality (AUC 0.77). Rubulotta et al [13] used a regression tree analysis to create a PIRO score and obtained a limited discriminatory performance (AUC 0.70). Rello et al [17] built a PIRO score from a cohort of 529 ICU patients admitted with community-acquired pneumonia. Their PIRO score showed very good discrimination (AUC 0.88) for known risks of community-acquired pneumonia. Lisboa et al [18] created a PIRO score for 441 ICU patients with ventilator-associated pneumonia. The ventilator-associated pneumonia PIRO score had a very good discrimination (AUC 0.81). Howell et al [16] built a derivation cohort to include 2132 patients to create a PIRO score. Their PIRO score also showed excellent discrimination (AUC 0.9). However, mortality in this cohort was very low as patients included came from the emergency department with “suspicion of infection” and patients with noninfectious diagnosis might also have been included.

We believe that our results show that the specific variables of each of the four domains are better suited than the variables from previous studies; in particular, we were able to select specific variables for each of the four components that may fit better to the consensus definition and concept of the PIRO.

All those previous studies present several limitations: restrict to a cohort of patients with a single cause of sepsis: community-acquired pneumonia or ventilator-associated pneumonia in the studies by Rello and Lisboa [17], [18]; based on secondary analysis of cohorts included on other studies with diverse aims as in the studies by Moreno and Rubollota [11], [13]; including patients with “suspicion of infection”, introducing the possibility that patients without sepsis might have been included, in particular if we look at the very low mortality rate in the study by Howell et al [16]. However, those previous studies have added substantial information: we understood that the PIRO concept may be brought into practice and that the PIRO concept might improve future research of sepsis. In cancer research, patients are not categorized only with “cancer”, they are also categorized as having a tumor that might be T3N0M0. This categorization has practical implications for decisions such as initiating or not chemotherapy/radiotherapy or any new drug as well as the timing and type of surgery. In sepsis patients, we need to move forward and begin to try new therapies for patients presenting with different PIRO scores.

We believe that the novel aspects developed in our study, i.e., the inclusion of biomarkers for the R component and a dynamic view of the patient daily evolution are a step forward in the building of the PIRO staging system.

The limitations from the present study include those pointed out on previous studies with the same cohort of patients [25], [26], [27], namely the exclusion of nosocomial infections and the need for validation in an independent population. Concerning the R component we only assessed one biomarker, CRP. It is possible that other biomarkers, namely procalcitonin, or even assessing panels of biomarkers might improve the results. Moreover, for antibiotic therapy we used the definition of concordant antibiotic therapy [38] as it was not possible to gather results according to adequacy of antibiotic therapy and microbiologic documentation was available in only 40% of the patients [25]. In addition, definitions for cancer disease was restricted to the definition for metastatic cancer used in the original SAPS II study [30] which may not reflect all the array of severity of cancer disease.

The present study has important strengths: it is the first step on trying to find a methodological way to describe a dynamic perspective on the PIRO staging system, with dynamic assessment of variables, an approach that may fit better to describe patients with sepsis as a dynamic process. Moreover, it is probably the first including more suitable variables for each of the four components of PIRO, in particular, the variables for the R component, which included the WCC and CRP in a dynamic view.

Currently, PIRO is still a research concept, not an established tool ready to be used at the bedside, however, with the present study we gave a step further in trying to find out which model with which variables may best fit on a tool able to give us, clinicians, information concerning the prognosis and treatment response of severe sepsis and septic shock and a step further on a future consensus. In conclusion, our results showed that this novel approach to PIRO concept present a good prediction of hospital mortality for patients with CAS admitted to ICUs. It is also our believe that PIRO system should be further investigated in order to become a true patient staging system with real treatment and prognostic implications in sepsis patients.

## Supporting Information

Table S1 **Evolution of clinical and laboratorial variables along the first five days in ICU.**
(DOCX)

Appendix S1 **SACiUCI Study Coordinator and list of all the participating ICUs and respective ICU study coordinator.**
(DOCX)
