# Supplementary material for: The Predisposition, Infection, Response and Organ Failure (Piro) Sepsis Classification System: Results of Hospital Mortality Using a Novel Concept and Methodological Approach
Source: PLoS One. 2013 Jan 18;8(1):e53885. doi: 10.1371/journal.pone.0053885 (PMC3548822; doi:10.1371/journal.pone.0053885)
Supplement: Table S1 — Evolution of clinical and laboratorial variables along the first five days in ICU. (DOCX) [file pone.0053885.s001.docx]

**Supplementary Table – Evolution of clinical and laboratorial variables along the first five days in ICU**

|  | **Total**  **(n=891)** | | **Non-survivors**  **(n=337)** | | **Survivors**  **(n=554)** | | **p-value*** |
| --- | --- | --- | --- | --- | --- | --- | --- |
| **Heart Rate D1,** mean ±SD | 116.0±24.3 | | 121.3±26.8 | | 112.8±22.1 | | <0.001** |
| **Heart Rate D2** | 111.1±24.8 | | 117.1±26.3 | | 107. 8±23.3 | | <0.001** |
| **Heart Rate D3** | 106.5±23.7 | | 113.6±24.5 | | 103.0±22.5 | | <0.001** |
| **Heart Rate D4** | 102.8±22.8 | | 108.4±25.2 | | 100.2±21.1 | | <0.001** |
| **Heart Rate D5** | 103.5±23.0 | | 109.6±27.0 | | 100.7±20.3 | | <0.001** |
| **WCC** (10^-3^*L)**D1,** median (IQR) | 13.2 (9.1-19.3) | | 13.8 (9.1-20.1) | | 12.9 (9.1-19.2) | | 0.825*** |
| **WCC D2** | 12.3 (9.0-17.7) | | 13.4 (9.6-19.5) | | 12.0 (8.9-16.4) | | 0.010*** |
| **WCC D3** | 11.9 (8.6-17.1) | | 12.8 (9.3-18.2) | | 11.2 (8.4-15.9) | | 0.001*** |
| **WCC D4** | 11.4 (8.3-15.9) | | 13.1 (8.7-17.9) | | 10.9 (8.1-15.2) | | 0.001*** |
| **WCC D5** | 11.4 (8.4-15.6) | | 12.1 (8.6-18.3) | | 11.2 (8.4-14.6) | | 0.024*** |
| **Neutrophils (%) D1,** median (IQR) | 89 (82-93) | | 89.0 (83-92) | | 88.9 (82- 93) | | 0.389*** |
| **Neutrophils D2** | 88 (82.6-92) | | 89 (85-93) | | 88 (82-92) | | 0.001*** |
| **Neutrophils D3** | 87 (81-91) | | 89 (84-93) | | 86 (79-90) | | <0.001*** |
| **Neutrophils D4** | 85 (78-90) | | 88.5 (82-92) | | 84 (76-89) | | <0.001*** |
| **Neutrophils D5** | 84 (76-90) | | 88 (82-92) | | 82 (75-89) | | <0.001*** |
| **C-reactive protein (mg/dL)D1,** mean ±SD | 20.1±12.6 | | 20.7±12.8 | | 19.8±12.5 | | 0.367** |
| **C-reactive protein D2** | 20.7±11.5 | | 21.8±11.4 | | 20.1±11.5 | | 0.059** |
| **C-reactive protein D3** | 18.4±11.0 | | 20.6±10.8 | | 17.3±11.0 | | <0.001** |
| **C-reactive protein D4** | 15.4±10.5 | | 18.4±10.9 | | 14.0±10.1 | | <0.001** |
| **C-reactive protein** **D5** | 13.1±9.7 | | 16.3±11.0 | | 11.6±8.6 | | <0.001** |
| **SOFA D1,** mean ±SD | 8.5±3.9 | | 10.3±4.0 | | 7.5±3.4 | | <0.001** |
| **SOFA D2** | 8,0±3.9 | | 10.0±4.1 | | 7.0±3.4 | | <0.001** |
| **SOFA D3** | 7,4±3.9 | | 9.3±4.1 | | 6.5±3.4 | | <0.001** |
| **SOFA D4** | 7,2±3.9 | | 9.4±4.0 | | 6.1±3.4 | | <0.001** |
| **SOFA D5** | 6,7±3.8 | | 9.0±4.1 | | 5.7±3.3 | | <0.001** |
| **Glycemia (mg/dL)D1** |  |  |  |  |  |  |  |
| <90 | 45 | | 27 | (60) | 18 | (40) |  |
| 90-150 | 448 | | 161 | (36) | 287 | (64) | 0.006 |
| >150 | 398 | | 149 | (37) | 249 | (63) |  |
| **Glycemia D2** |  |  |  |  |  |  |  |
| <90 | 29 | | 16 | (55) | 13 | (45) |  |
| 90-150 | 505 | | 173 | (34) | 332 | (66) | 0.015 |
| >150 | 357 | | 148 | (41) | 209 | (59) |  |
| **Glycemia D3** |  |  |  |  |  |  |  |
| <90 | 21 | | 7 | (33) | 14 | (67) |  |
| 90-150 | 466 | | 142 | (30) | 324 | (70) | <0.001 |
| >150 | 404 | | 188 | (47) | 216 | (53) |  |
| **Glycemia D4** |  |  |  |  |  |  |  |
| <90 | 17 | | 6 | (35) | 11 | (65) |  |
| 90-150 | 467 | | 135 | (29) | 332 | (71) | <0.001 |
| >150 | 407 | | 196 | (48) | 211 | (52) |  |
| **Glycemia D5** |  |  |  |  |  |  |  |
| <90 | 14 | | 6 | (43) | 8 | (57) |  |
| 90-150 | 439 | | 127 | (29) | 312 | (71) | <0.001 |
| >150 | 438 | | 204 | (47) | 234 | (53) |  |
| **Lactate(mmol/L) D1** |  |  |  |  |  |  |  |
| <=2 | 377 | | 99 | (26) | 278 | (74) |  |
| >2 | 509 | | 236 | (46) | 273 | (54) | <0.001 |
| **Lactate D2** |  |  |  |  |  |  |  |
| <=2 | 423 | | 115 | (27) | 308 | (73) |  |
| >2 | 463 | | 221 | (48) | 242 | (52) | <0.001 |
| **Lactate D3** |  | |  |  |  |  |  |
| <=2 | 440 | | 121 | (28) | 319 | (73) |  |
| >2 | 442 | | 210 | (48) | 232 | (52) | <0.001 |
| **Lactate D4** |  | |  |  |  |  |  |
| <=2 | 438 | | 120 | (27) | 318 | (73) |  |
| >2 | 449 | | 216 | (48) | 233 | (52) | <0.001 |
| **Lactate D5** |  | |  |  |  |  |  |
| <=2 | 441 | | 117 | (27) | 324 | (73) |  |
| >2 | 449 | | 220 | (49) | 229 | (51) | <0.001 |
| **Lactate D1** |  |  |  |  |  |  |  |
| <=4 | 602 | | 190 | (32) | 412 | (68) |  |
| >4 | 287 | | 146 | (51) | 141 | (49) | <0.001 |
| **Lactate D2** |  |  |  |  |  |  |  |
| <=4 | 628 | | 193 | (31) | 435 | (69) |  |
| >4 | 263 | | 144 | (55) | 119 | (45) | <0.001 |
| **Lactate D3** |  |  |  |  |  |  |  |
| <=4 | 602 | | 189 | (31) | 413 | (69) |  |
| >4 | 289 | | 148 | (51) | 141 | (49) | <0.001 |
| **Lactate D4** |  |  |  |  |  |  |  |
| <=4 | 572 | | 177 | (31) | 395 | (69) |  |
| >4 | 318 | | 160 | (50) | 158 | (50) | <0.001 |
| **Lactate D5** |  |  |  |  |  |  |  |
| <=4 | 548 | | 167 | (30) | 381 | (70) |  |
| >4 | 343 | | 170 | (50) | 173 | (50) | <0.001 |

HCAS – Health-care associated sepsis; SAPS II – Simplified Acute Physiology Score II; MDR –Multi-drug resistant

microorganism; *Chi-square test, ** t Student test, *** Mann-Whitney test, IQR – interquartile range,

SD – standard deviation
