# Supplementary material for: The Predisposition, Infection, Response and Organ Failure (Piro) Sepsis Classification System: Results of Hospital Mortality Using a Novel Concept and Methodological Approach
Source: PLoS One. 2013 Jan 18;8(1):e53885. doi: 10.1371/journal.pone.0053885 (PMC3548822; doi:10.1371/journal.pone.0053885)
Supplement: Appendix S1 — SACiUCI Study Coordinator and list of all the participating ICUs and respective ICU study coordinator. (DOCX) [file pone.0053885.s002.docx]

**APPENDIX**

*Study Coordinator.* António H. Carneiro (Unidade de Cuidados Intensivos Polivalente, Hospital Geral de Santo António, Porto). *Study Promoters.* António H. Carneiro (Unidade de Cuidados Intensivos Polivalente, Hospital Geral de Santo António, Porto), Eduardo Silva (Unidade de Cuidados Intensivos Polivalente, Hospital do Desterro, Lisboa), and José Artur Paiva (Unidade de Cuidados Intensivos Polivalente da Urgência, Hospital de São João, Porto).

*Participating Intensive Care Units.* Unidade de Cuidados Intensivos Polivalente, Hospital Geral de Santo António, Porto (Teresa Cardoso), Unidade de Cuidados Intensivos Polivalente da Urgência, Hospital de São João, Porto (José Manuel Pereira), Unidade de Cuidados Intensivos,

Instituto Português de Oncologia, Porto (Filomena Faria), Unidade de Cuidados Intensivos Médicos, Hospital Pedro Hispano, Matosinhos (António Sarmento, Luís Ribeiro), Unidade de Cuidados Intensivos, Centro Hospitalar de Vila Nova de Gaia, Gaia (Paula Castelões), Unidade

de Cuidados Intensivos, Hospital da Senhora da Oliveira, Guimarães (Anabela Bártolo), Unidade de Cuidados Intensivos, Hospital de São Pedro, Vila Real (Francisco Esteves), Unidade de Cuidados Intensivos, Hospital de São Sebastião, Santa Maria da Feira (Piedade Amaro), Serviço de Medicina Intensiva, Hospitais da Universidade de Coimbra, Coimbra (Paulo Martins), Unidade de Cuidados Intensivos, Centro Hospitalar de Coimbra, Coimbra (Paula Coutinho), Unidade de Cuidados Intensivos Polivalente, Hospital do Desterro, Lisboa (Paulo Gomes), Serviço de Medicina Intensiva, Hospital de Santa Maria, Lisboa (Henrique Bento), Unidade de Urgência Médica, Hospital de São José, Lisboa (Luís Reis), Unidade de Cuidados Intensivos Médicos, Hospital de São Francisco Xavier, Lisboa (Pedro Póvoa), Unidade de Cuidados Intensivos Médico-Cirúrgica, Hospital Pulido Valente, Lisboa (Armindo Ramos), Unidade de Cuidados Intensivos, Hospital José Joaquim Fernandes, Beja (José Vaz), and Unidade de Cuidados Intensivos, Centro Hospitalar do Barlavento Algarvio, Portimão (Carlos Glória).
